# Telephone versus web panel National Survey for monitoring adoption of preventive behaviors to climate change in populations: a case study of Lyme disease in Québec, Canada

**DOI:** 10.1186/s12874-020-00958-4

**Published:** 2020-04-10

**Authors:** Grâce Ngambo Domche, Pierre Valois, Magalie Canuel, Denis Talbot, Maxime Tessier, Cécile Aenishaenslin, Catherine Bouchard, Sandie Briand

**Affiliations:** 1 Faculty of Education, Université Laval, 2320, rue des Bibliothèques, Quebec City, QC G1V 0A6 Canada; 2 National Institute of Public Health of Québec, 945 av Wolfe, Quebec City, QC G1V 5B3 Canada; 3 Faculty of Medicine, Université Laval, 1050 Avenue de la Médecine, Quebec City, QC G1V 0A6 Canada; 4 Populational health and optimal practices axis, CHU de Québec – Université Laval Research center, Quebec City, QC G1S 4L8 Canada; 5 Groupe de recherche en épidémiologie des zoonoses et santé publique (GREZOSP), Faculté de médecine vétérinaire (FMV), Université de Montréal, Saint-Hyacinthe, QC Canada; 6 Public Health Risk Sciences Division, National Microbiology Laboratory, Public Health Agency of Canada, Saint-Hyacinthe, QC Canada; 7 National Institute of Public Health of Québec, Montréal, QC Canada

**Keywords:** Web survey, Telephone survey, Lyme disease, Climate change, Adaptation

## Abstract

**Background:**

To monitor the adoption of climate change adaptive behaviors in the population, public health authorities have to conduct national surveys, which can help them target vulnerable subpopulations. To ensure reliable estimates of the adoption of these preventive behaviors, many data collection methods are offered by polling firms. The aim of this study was to compare a telephone survey with a web survey on Lyme disease with regard to their representativeness.

**Methods:**

The data comes from a cross-sectional study conducted in the Province of Québec (Canada). In total, 1003 people completed the questionnaire by telephone and 956 filled in a web questionnaire. We compared the data obtained from both survey modes with the census data in regard to various demographic characteristics. We then compared the data from both samples in terms of self-reported Lyme disease preventive behaviors and other theoretically associated constructs. We also assessed the measurement invariance (equivalence) of the index of Lyme disease preventive behaviors across the telephone and web samples.

**Results:**

Findings showed that neither the telephone nor the web panel modes of data collection can be considered more representative of the target population. The results showed that the proportion of item non-responses was significantly higher with the web questionnaire (5.6%) than with the telephone survey (1.3%), and that the magnitude of the differences between the two survey modes was nil for 19 out of the 30 items related to Lyme disease, and small for 11 of them. Results from invariance analyses confirmed the measurement invariance of an index of adaptation to Lyme disease, as well as the mean invariance across both samples.

**Conclusions:**

Our results suggested that both samples provided similar estimates of the level of adaptation to Lyme disease preventive behaviors. In sum, the results of our study showed that neither survey mode was superior to the other. Thus, in studies where adaptation to climate change is monitored over time, using a web survey instead of a telephone survey could be more cost-effective, and researchers should consider doing so in future surveys on adaptation to climate. However, we recommend conducting a pretest study before deciding whether to use both survey modes or only one of them.

## Background

With the unavoidable warming of the climate system more frequent climatic hazards, such as heat waves and floods, will result in greater health impacts on the population [1]. For instance, higher heat exposure due to average temperature warming, as well as heat waves and urban heat islands, affects the health of organizations, systems, and populations [2–4]. Heat also affects the health of populations by causing, for instance, increased air pollution, the spread of disease vectors, food insecurity, and undernutrition [5]. The impacts of the changing climate on human health include not only these dangerous climatic events, but also a probable increase in the occurrence zoonotic diseases. One example is Lyme disease, a tick-borne zoonosis which occurs mostly in temperate regions. Its vector, *Ixodes scapularis* tick, is slowly spreading further north due to rising global temperatures and are now found in areas where they never were before and the disease affects an increasing number of Canadians since the last decade [6–13]. The Public Health Agency of Canada is reporting an increase of over 588% in reported cases in Canada since 2009 [14]. In Québec, where Lyme disease is a notifiable disease, there were 338 cases of Lyme disease contracted in 2019, compared to 66 in 2014 [15].

Because of these deleterious effects of global warming and climate change on human health, various national surveys are being conducted to determine whether the preventive behaviors promoted by public health authorities are adopted by the population. These surveys are used to monitor adaptation to climate change over time. They enable public health agencies to better identify protective measures that should be reinforced in health promotion campaigns and to target groups that are not adapting.

The results of these surveys are based on sampling procedures intended to generate representative samples of the populations concerned with specific effects of climate change. Over the past four decades, telephone surveys were one of the data collection methods favored by researchers in psychology, sociology, and health-related fields to monitor behaviors, health status, and other determinants [16, 17]. However, in recent years, telephone surveys have gradually been supplanted by methods incorporating computer technologies [17]; for a more detailed discussion of these developments, see [18]. The use of web surveys has increased and expanded quickly around the world [19–21] in large part because of the extremely low marginal cost for each additional case in a web survey compared with a telephone survey [17]. At the Québec Observatory of Adaptation to Climate Change, we carried out several surveys in recent years. Our experiences with the survey firms that we contacted to submit proposals to administer our questionnaires indicate that low cost is definitely one of the greatest benefits of web surveys over telephone surveys.

Recent studies showed that web panel surveys would have additional advantages over telephone surveys besides the lower cost, for instance: the absence of interviewer bias [22], the fact that a self-administered questionnaire makes the respondents more open and honest [23–28], and the possibility of completing the questionnaire at one’s own pace [22, 29]. However, web panel surveys present some non-negligible disadvantages compared with telephone surveys: they do not include the portion of the population without an internet connection [30], one of the main reasons why most web samples are generally considered non-probabilistic [31]; response rates are usually lower than with telephone surveys [17, 32]; and the respondents tend to be younger, to be more educated, and to have higher incomes than non-internet users [33–35]. Finally, other disadvantages include the absence of interviewers to explain the meaning of a question to the respondents, and the impossibility of recruiting individuals with problems of illiteracy or blindness [22].

Overall, some researchers agree that the web and telephone modes present dissimilarities that cannot be easily dismissed, whereas others are more inclined to believe that both modes are relatively similar and can even complement each other [36, 37]. The legitimacy of these two points of view is undoubtedly subject to the context of the surveys conducted. Furthermore, the differences or similarities observed between the data collected via these two modes may depend on the topic of the study. For example, in the case of condom use, one can expect a greater risk of the effect of social desirability in data collected by phone than via the web [38, 39]. Inversely, a telephone interview could be expected to produce more reliable results in a study concerning living wills. Indeed, in such a case, the interviewer could help reduce measurement errors by providing respondents with explanations of the specific meaning of questions that refer to a more abstract concept like living will.

A sample generated in web mode can be considered either probabilistic or non-probabilistic. In a non-probabilistic web panel mode of data collection, the sample consists of volunteers recruited by responding favorably to prior invitations. Those invitations are usually made through popular websites, internet portals, or other means [40, 41]. In a probabilistic web panel mode, participants are selected at random with a procedure ensuring that all the people in the population have equal or known probabilities of being chosen, as in the case of random digit dialing (RDD). Phone numbers are generated at random and people reached are subsequently invited to join the web panel for a survey [22].

Our research team (Québec Observatory for the Adaptation to Climate Change) conducts surveillance of the adoption of climate change adaptation behaviors in the province of Québec, Canada (1,667,441 km^2^, or 643,802 mile^2^; population of 8.3 million). Several surveys are conducted annually and must be replicated over time to monitor the evolution of adaptation. It is important to determine from the outset which data collection mode is best for future surveys. This study is part of a more general project aiming to identify the factors that are associated with the adoption of Lyme disease prevention behaviors (LDPB). It pertains more specifically to the issue of modes of information collection and seeks to detect potential differences between two samples of respondents contacted either via the web (non-probabilistic sample) or by phone (probabilistic sample). The differences sought concern not only the results observed but also the representativeness of the two samples in terms of certain characteristics of the target populations. Both survey modes are often proposed by polling firms in the Province of Québec (Canada), where the household rate of internet connection was estimated at 90% in 2016 [42].

The three specific objectives of this study are to: (1) compare the representativeness of the two samples with census data in terms of sociodemographic characteristics (gender, age, highest level of education, annual income, presence of children in the household, household size), (2) compare these survey modes with regard to the estimation of the population’s rates of reported Lyme disease adaptive behaviors and other theoretically associated variables, and (3) test the measurement invariance or equivalence of the latent construct of Lyme disease adaptive behavior across the non-probabilistic web panel and telephone samples. For instance, a non-invariance of the uniquenesses of the behavioral items composing the latent construct of Lyme disease adaptation would indicate that this construct is assessed with different levels of measurement error and precision in the two survey modes. Consequently, health agencies’ decisions to protect individuals against Lyme disease would depend on the survey mode chosen, which is not desirable. It is also possible to use weighting methods to correct for the non-representativeness of the samples as compared to census data. Thus, comparisons of the data obtained from the telephone and web surveys will also be performed on the weighted data for all three objectives to verify the effect of the statistical correction.

## Methods

### Target population

The data comes from a cross-sectional study conducted in the Province of Québec (Canada). The target population studied consisted of people living in a municipality where the risk of contracting Lyme disease was low (i.e. where at least one tick was collected through active surveillance activities) or significant (i.e. at least three human cases of Lyme disease acquired locally in a municipality of fewer than 100,000 inhabitants or at least 23 tick submissions from human passive surveillance acquired in the municipality in the past 5 years in a municipality of fewer than 100,000 inhabitants, or three life-cycle stages—larva, nymph, and adult—of the tick collected in the municipality in 1 year, through active surveillance in which at least one nymph tested positive for *Borrelia burgdorferi*). Most of the municipalities where the risk was significant were located in the southern part of the province [43]. The respondents had to be at least 18 years old and speak English or French to participate in the study.

We used the 2016 Canadian census data to compare the representativeness of the web and telephone samples in terms of sociodemographic characteristics (gender, age, highest level of education, annual income, presence of at least one child in the household, household size). Census statistics specific to our target population were obtained from Statistics Canada (at the cost of CAD 1400), for people 18 years of age and older living in one of the 111 municipalities that were included in our study.

The census data came from two questionnaires. One was a short-form questionnaire addressed to 100% of the Canadian population, which provided information on age, gender, household size, and family structure. The response rate was very high, at 98.4%, which represents 15,067,444 private dwellings [44]. The census data for our target population covered 1,031,220 private dwellings, or 1,924,155 people aged 18 years or older.

The other questionnaire was a long-form questionnaire administered to 25% of Canadian private dwellings (hereafter named “25% census sample”), which included information on the highest education level and the total household income. The response rate was also very high, at 97.8%, the best ever recorded [45]. Given this high response rate, we decided to keep this data subset to determine the level of representativeness of the web and telephone samples in regard to the highest education level and the annual income. This data subset was weighted according to the sampling probability of being selected; see Statistique Canada [46] for more details regarding the weighting method used.

### Samples and data collection

For the web survey, a polling firm (BIP Research) surveyed 956 people (women, 55.4%) through a web questionnaire. The data were collected from May 18 to August 28, 2018. The web sample was selected from a web panel that included 40,000 Quebeckers at least 18 years old, all randomly recruited by telephone from previous probabilistic surveys. Of the 40,000 people, 5512 lived in the municipalities where the risk of contracting Lyme disease was low or significant and were contacted for our study. The municipalities of residence were verified a second time with a question in the online questionnaire. The respondents were not paid to participate in the survey, but the survey firm organizes a lottery each quarter (i.e., four times per year), so the members of the panel who answered a questionnaire during this period of time have the chance of winning CAD 1000. A panel member is not solicited more than six times a year. New panel members are recruited every week, and inactive members are removed from the database. According to the survey firm, the average panel recruitment response rate at the time of the current study is between 20 and 30%, depending on the subject and the length of the questionnaire.

For the telephone sample, the polling firm randomly drew 200,000 of all the available landline phone numbers. Among those drawn, 64,631 corresponded to the target municipalities. Of the 64,631 numbers, 6985 were called, and the person was asked to participate in the present study. The data from this sample were also collected from May 18 to August 28, 2018. A maximum of 10 attempts were made to establish contact before a telephone number was rejected. A total of 1003 people (woman, 68.3%) completed the questionnaire by telephone.

We used the Kish selection method to determine quotas by region [47, 48]. The quotas for smaller and larger regions were readjusted to ensure minimal statistical power in smaller regions and to meet the quota of 1000 respondents for both samples (see online resource 1 for the quotas and online resource 2 for the final samples).

We used Kish’s [49] formula to estimate the effective sample size (ESS) of both web questionnaire and telephone samples, after weighting the data. The ESS estimates the “worth” of a given sample as compared to a simple random sampling from the population. The ESS of the 956 people surveyed by the web questionnaire was 383, whereas that of the 1003 people who answered the telephone questionnaire was 238.

### Questionnaire

Participants were asked to complete a 91-item questionnaire administered as part of a larger survey conducted by the Québec Observatory of Adaptation to Climate Change. The aim of this survey was to identify factors associated with the adoption of LDPB among people in the Province of Québec (Canada). Of these questions, 30 were linked to psychosocial models, in particular to the theory of planned behavior and the health belief model, and were used in the current study to compare the telephone and web data collection modes. The questions pertained to Lyme disease exposure (three items), knowledge of the disease (three item), risk perception (two items), vulnerability (one item), opinions on vaccines (three items), attitudes towards the adoption of LDPB (one item), perception of social pressure (one item), perception of control over these behaviors (one item), intention to adopt these behaviors (one item), and various (14 items) self-reported adopted LDPB (Table 1).

**Table 1:** Description of the variables measured

Variables	Questions/Items	Response options
**Lyme disease exposure**		
	• Have you ever found a tick on yourself? • Have you ever been bitten by a tick? • Has a doctor ever diagnosed you with Lyme disease?	1 = No 2 = Yes 3 = Uncertain (I think so / I don’t think so, but I’m not sure)
**Knowledge of the disease**		
	• Before responding to this survey, had you ever heard of Lyme disease?	1 = No 2 = Yes
	• I am going to read you four descriptions. Please tell me which one you believe best describes Lyme disease.	1 = It’s a disease transmitted through contact with other people 2 = It’s a disease transmitted through tick bites (right answer) 3 = It’s a disease transmitted through saliva 4 = It’s a disease transmitted through a dog bite
	• Based on your current knowledge, the first symptom of Lyme disease is generally	1 = Diarrhea 2 = Vomiting 3 = A red plaque on the skin (right answer) 4 = Nasal congestion 5 = A persistent cough
**Risk perception**		
	• In your opinion, what is the risk of you contracting Lyme disease in the next year? Would you say that it is:	1 = Nil 2 = Very low 3 = Low 4 = Moderate 5 = High 6 = Very high
	• Do you believe that it is possible to contract Lyme disease in your municipality?	1 = No 2 = Yes
**Vulnerability**		
	• If you were to contract Lyme disease, would you say that the consequences for your health would be very serious?	1 = No, not at all 2 = No, not really 3 = Yes, mostly 4 = Yes, absolutely
**Opinions on vaccines**		
	• If a vaccine against Lyme disease were available, you would get vaccinated. • If a vaccine against Lyme disease were available, you would get your child vaccinated • Vaccines are a danger to your health. Do you:	1 = Strongly disagree 2 = Somewhat disagree 3 = Somewhat agree 4 = Strongly agree
**Theory of planned behavior constructs**		
**Attitudes towards adopting behaviors**	• Adopting behaviors that will protect you against Lyme disease in the next year will be:	1 = Very useless 2 = Slightly useless 3 = Slightly useful 4 = Very useful
**Perceived behavioral control**	• Do you agree that it will be easy to protect yourself against Lyme disease in the next year?	1 = Strongly disagree 2 = Somewhat disagree 3 = Somewhat agree 4 = Strongly agree
**Perceived social norms**	• If you adopt behaviors to protect yourself against tick bites and therefore Lyme disease in the next year, people who are important to you will support your choice.	1 = Strongly disagree 2 = Somewhat disagree 3 = Somewhat agree 4 = Strongly agree
**Behavioral intentions**	• You intend to adopt behaviors to protect yourself against tick bites and Lyme disease in the next year.	1 = Strongly disagree 2 = Somewhat disagree 3 = Somewhat agree 4 = Strongly agree
**Preventive behaviors**^**a**^		
	• Has ever looked into ways to prevent Lyme disease? • Have you ever looked into the potential consequences of Lyme disease for your physical or mental health?	0 = No 1 = Yes
	• When practicing outdoor activities, do you wear long pants and a long-sleeved sweater? • When practicing outdoor activities, do you wear closed shoes? • When practicing outdoor activities, do you tuck the bottom of your sweater or of your shirt into your pants? • When practicing outdoor activities, do you tuck the bottom of your pants into your socks or boots? • When outdoors, do you use a bug repellent (containing DEET, icaridin, or picaridin) on your clothes or the exposed parts of your body? • When practicing outdoor activities, do you walk on cleared paths and trails, avoiding tall grass? • When practicing outdoor activities, do you wear light-colored clothing to make it easier to check for ticks? • After being outdoors, do you examine your body for ticks and remove them immediately? • After being outdoors, do you examine your clothes and the items that you had with you to avoid bringing ticks into your home? • After being outdoors, do you put your clothes in the dryer for six minutes to eliminate ticks that may be there?	1 = Never 2 = Rarely 3 = Occasionally 4 = Often 5 = Always
	• Do you regularly mow your lawn or have it mown?	1 = I don’t have a lawn 2 = No 3 = Yes, less than once a week 4 = Yes, about once a week 5 = Yes, more than once a week
	• How often do you maintain your lawn, for example pick up dead leaves, weeds, branches or twigs, or have them picked up? (other than mowing your lawn)	1 = Never 2 = Less than once a month 3 = Once or twice a month 4 = Once a week 5 = More than once a week

^a^ All of these preventive behaviors were used to create and validate an index of LDPB

The self-reported adopted preventive behaviors were selected based on a literature review [50–55] and recommendations from public health agencies [43, 56]. Using these behavioral indicators, we created and validated an index of LDPB [57]. The index was validated for both the telephone interviews and the web self-administered questionnaire.

These questions and their respective response options are presented in Table 1.

### Statistical analysis

First, to assess the representativeness of the telephone and web surveys, we compared the data obtained through each data collection mode with the census data in regard to gender, age, presence of children in the household, and household size. We also compared the telephone and web surveys with the 25% census sample in regard to annual income and highest education level. To do so, we used a one-sample chi-square test or a Fisher’s exact test (when the minimum number of observations in a cell was less than 5) and evaluated the effect size using Cramer’s V statistic.

Second, we compared the data collected from the telephone and web surveys on specific items of the questionnaire in regard to means and proportions of self-reported Lyme disease adaptive behaviors and other theoretically associated constructs. A Student’s t-test or chi-square test/Fisher’s exact test was used depending on whether means or frequencies were being compared. Z tests for independent proportions were also performed on all the categories of nominal variables. The effect sizes were also calculated: Cohen’s *d* for Student’s t-tests, Cramer’s V for chi-square/Fisher’s exact tests, and Cohen’s *h* for the Z tests for independent proportions [58]. Table 2 provides a better understanding of how to interpret those effect size indicators. In addition, we compared the web and telephone data on the nonresponse rates for each of the items mentioned earlier, as well as the sociodemographic variables. The nonresponse rates consisted of all those who answered “Do not know” and those who simply refused to give an answer. We used Z tests for independent proportions for these comparisons.

**Table 2:** Thresholds for the interpretation of Cohen’s d and Cramer’s V

Indices	Effect size			
	Nil	Small	Medium	Large
Cohens’ d and h	[0.0–0.2[	[0.2–0.5[	[0.5–0.8[	[0.8-∞[
Cramer’s V by degrees of freedom				
1	[0.0–0.10[	[0.10–0.30[	[0.30–0.50[	[0.50-∞[
2	[0.0–0.07[	[0.07–0.21[	[0.21–0.35[	[0.35-∞[
3	[0.0–0.06[	[0.06–0.17[	[0.17–0.29[	[0.29-∞[
4	[0.0–0.05[	[0.05–0.15[	[0.15–0.25[	[0.25-∞[
5	[0.0–0.05[	[0.05–0.13[	[0.13–0.22[	[0.22-∞[

Third, we assessed the measurement invariance (equivalence) of the index of LDPB across the telephone and web samples. This invariance is a necessary condition for unambiguous mean comparisons of the index across both survey modes [59–61].

First, a model with no invariance of any parameters, also referred to as the configural invariance model, was estimated. We then tested the strong invariance of the model by constraining the factor loadings and item thresholds to equality for both groups, which is equivalent to the weak invariance model because we used binary items [62]. Third, we tested the strict invariance of the model by constraining the factor loadings, item thresholds, and item uniquenesses to equality across groups. Finally, we tested the invariance of the latent variance and latent mean of the estimated factor. In this study, we used the comparative fit index (CFI), the Tucker-Lewis index (TLI), and the root mean squared error of approximation (RMSEA) to compare models and assess model fit. Acceptable model fit is indicated by CFI and TLI values greater than or equal to 0.90 and less than 0.95. Excellent model fit is indicated by CFI and TLI values greater than or equal to 0.95. As for RMSEA, values between 0.05 and 0.08 indicate an adequate model fit, and values less than or equal to 0.05 indicate an excellent model fit [63, 64].

Finally, we reproduced the previous statistical analyses where the data collected from the telephone and web surveys were compared once the data were weighted for both samples. To do so, we first weighted the data by administrative region with a raking ratio method using six variables: age, sex, household size, household structure, highest education level, and income [65]. Second, we reweighted the data using a ratio adjustment, so that the proportions of the administrative regions in the web and telephone samples would be the same as those in the population.

## Results

The response rates for the telephone and web surveys were 24.5, and 17.0%, respectively. Telephone response rate was determined by dividing the number of completed questionnaires by the number of eligible individuals. We also took into account a proportion of the cases of unknown eligibility in the number of eligible individuals, as indicated in the American Association for Public Opinion Research Standard Definitions [66]. The proportion of cases of unknown eligibility taken into account was estimated by dividing the number of valid telephone numbers by the number of valid telephone numbers added to the number of invalid telephone numbers. For the web survey, the response rate was determined by dividing the number of completed questionnaire by the number of sent emails.

In the web survey, 44.6% of the respondents were men and 55.4% were women, compared with 31.7 and 68.3%, respectively, in the telephone survey. The average connection time to the website was 17 min in the web survey, whereas the telephone interviews lasted an average of about 20 min.

### Comparison of the web and telephone surveys with the census

The results presented in Table 3 showed that, in general, the percentages of non-responses for sociodemographic variables were very low and similar (no effect size) in both surveys, except for the reported household annual gross income were there is a higher percentage of non-responses for the reported household annual gross income in the telephone survey (15.8% for the web sample and 20.5% for the telephone sample). This difference was statistically significant but negligible according to effect size analyses (Cohen’s h < .20).

**Table 3:** Item Non-Responses for the Sociodemographic Variables in the Web and Telephone Surveys

Sociodemographic variables	% of item non-responses		*Z* test	Cohen’s h^a^
	Web	Phone		
Age	No missing data	No missing data	n/a	0
Gender	No missing data	No missing data	n/a	0
Highest education level	0.7	0.6	−0.37	0.02 ^ne^
Household size	1.5	0.5	−2.18*	0.10 ^ne^
Household annual gross income	15.8	20.5	2.72**	0.12 ^ne^
Presence of at least one child in the household	2.0	0.8	−1.53	0.07 ^ne^

*p < .05; **p < .01

^a^ Effect size interpretation: ^ne^ No effect, ^†^ Small effect, ^††^ Moderate effect, ^†††^ Large effect

Our results showed also that almost all the sociodemographic characteristics of the web and telephone surveys were statistically different from those in the census and the 25% census sample (see Table 4). The only two exceptions were the average household annual gross income obtained in the web survey, where the distribution appeared to be representative of the population, and the presence of at least one child in the household, where the estimates in the telephone survey were not statistically different from the census data. Results regarding the magnitude or practical significance of these differences showed that the effect size for the web survey could be qualified as medium for two variables (age and highest education level), small for two other variables (household size and presence of at least one child in the household), and nil for one variable (gender). For the telephone survey, the results indicated one large (age), two medium (gender and highest education level), and two small (household size and household annual gross income) effect sizes.

**Table 4:** Comparison of the Telephone and Web Survey Data with the Census Data Regarding Sociodemographic Variables

Sociodemographic variables	Census	Sample		Chi-square test (χ^2^) and effect size (Cramer’s V)^a^					
		Web	Phone	Web-Census		Telephone-Census		Web-Telephone	
				χ^2^	Cramer’s V	χ^2^	Cramer’s V	χ^2^	Cramer’s V
**Age**									
• 18–34	25.6%	11.3%	5.6%	178.76****	0.19^††^	312.07****	0.25^†††^	35.66***	0.14^†^
• 35–44	16.4%	13.9%	14.4%						
• 45–54	18.8%	19.1%	17.7%						
• 55–64	18.6%	27.8%	26.4%						
• 65–74	12.9%	21.3%	24.0%						
• 75 and more	7.7%	6.6%	11.9%						
**Gender**									
• Male	48.5%	44.6%	31.7%	5.97*	0.08	113.40****	0.34^††^	34.34***	−0.13^†^
• Female	51.5%	55.4%	68.3%						
**Household size**									
• 1 person	30.5%	25.8%	22.4%	58.32****	0.18^†^	40.25****	0.14^†^	7.69*	0.06
• 2 persons	34.2%	44.1%	41.7%						
• 3 persons and more	35.3%	30.1%	35.9%						
**Presence of at least one child in the household**									
• Yes	39.2%	28.7%	36.3%	43.53****	0.22^†^	3.54 ^n.s.^	0.06	12.68***	−0.08
• No	60.8%	71.3%	63.7%						
**Highest education level**^**b**^									
• No certificate, diploma, or degree	17.4%	8.1%	12.1%	165.57****	0.24^††^	240.86****	0.28^††^	19.33***	0.10^†^
• Secondary (high) school diploma or equivalency certificate	23.3%	29.2%	30.2%						
• Diploma or certificate of college, trade, or vocational studies, or partial university studies	39.3%	30.7%	23.1%						
• University degree	20.0%	32.0%	34.6%						
**Household annual gross income**^**b**^									
• $20,000 and less	9.7%	9.2%	12.2%	6.24^n.s.^	0.05	10.60*	0.07^†^	13.40**	0.09
• $20,001–$60,000	37.1%	41.1%	37.6%						
• $60,001–$100,000	26.3%	27.7%	22.7%						
• More than $100,000	26.9%	22.0%	27.5%						

*p < .05. **p < .01. ***p < .001 ****p < .0001

^a^ Effect size interpretation: ^†^ Small effect, ^††^ Moderate effect, ^†††^Large effect

^b^25% census sample

Examination of Table 4 also revealed that the differences between the web and the telephone samples were all statistically significant. Furthermore, although the differences for the household annual gross income, the presence of at least one child in the household, and the household size were statistically significant, the magnitude of these differences was negligible according to the effect size analysis. The results also indicated that the effect sizes were small for age (Cramer’s V = 0.14), gender (Cramer’s V = 0.13), and the highest education level obtained (Cramer’s V = 0.10).

According to Z tests for independent proportions performed for every category of the sociodemographic variables, there was a significantly greater proportion of 18- to 34-year-olds participating in the web survey than in the telephone survey (Z = 4.51, *p* < .0001). However, this proportion for the web survey was still significantly lower than that for the census (Z = 10.18, *p* < .0001). There was also a smaller proportion of people aged 75 years or more in the web survey (Z = 4.00, *p* < .0001). The web survey proportion for this age category is not different to the proportion in the census (Z = − 1.24, *p* = 0.2143), whereas it is for the telephone survey (Z = 4.99, *p* < .0001). The results also showed a greater proportion of males in the web survey (Z = 5.86, *p* < .0001) than in the telephone survey, both proportions being smaller than the census proportion (Z = − 2.44, *p* = .0146, Z = − 10.65, *p* < .0001, respectively). Furthermore, compared to the telephone survey, there was a greater proportion of participants with a diploma or certificate of college, trade, or vocational studies, or with partial university studies (Z = 3.78, *p* < .0002), and a smaller proportion of participants with no certificate, diploma, or degree in the web survey (Z = 2.93, *p* < .0033).

We reproduced the previous statistical analyses using the weighted data for both samples. As expected, the results showed no significant difference between the census data and the web survey or between the census data and the telephone survey in terms of the proportions observed for each of the sociodemographic variables (see online resource 3).

### Comparison of the web and telephone surveys regarding items related to Lyme disease

Chi-square tests were used to test for differences between the web and the telephone questionnaires in the proportions of item non-response for each variable. Results showed that the global proportion of item non-response was significantly higher with the web questionnaire (5.6%: 54/956) than with the telephone survey (1.3%: 12/1003): χ^2^ = 26.02(1), *p* < 0.00001. Taken individually, the item non-response was also higher for the web questionnaire in the majority of the variables tested (25 out of 30) (see online resource 4). The 14 questions related to preventive behaviors had the lowest item non-response rates. Furthermore, χ^2^ tests or Fisher’s exact tests (FET) indicated that there were no statistical differences between the web and telephone surveys for four preventive behaviors. The results also showed no effect size for 12 behaviors, and a small effect size for two behaviors. The questions with the highest non-response rates were related to the respondents’ knowledge of the disease, their perceptions of the risk of contracting Lyme disease in their municipality, their perception of vulnerability or severity if they were to contract the disease, their opinions about vaccination, and psychosocial determinants of their own anticipated behaviors in the next year to protect themselves against Lyme disease (e.g. their attitudes towards the adoption of Lyme disease prevention behaviors in the next year). Cramer’s V tests indicated that among this subset of 16 variables theoretically related to the adoption of preventive behaviors to protect oneself against Lyme disease, the magnitude of the differences between the web and telephone surveys was nil for five questions, small for 10 questions, and medium for one question (perception of the risk of contracting Lyme disease in their municipality).

We also compared the statistical weighted estimates (proportions) obtained from the web and telephone surveys for the 30 questions (i.e., 14 preventive behaviors and 16 theoretically related variables) using the chi-square or the Fisher’s exact tests. The results showed that the weighted proportions in the web and telephone surveys were significantly different for all three of the questions related to Lyme disease exposure (Table 5). The magnitude of these differences is negligible for one of these three questions (ever been bitten by a tick) and small for two of the three questions (diagnosed with Lyme disease by a doctor, and found a tick on themselves).

**Table 5:** Comparison of weighted item responses from the Web and Telephone Surveys on Lyme Disease Exposure

Type of variables	%		χ^2^ test or Fisher exact test (FET)^a^	Cramer’s V^b^
	Web	Phone		
**Found a tick on yourself?**				
• Yes	5.4	4.8	χ^2^ = 23.21, p < .001	0.11^†^
• No	90.3	94.3		
• Uncertain	4.3	10.9		
**Ever been bitten by a tick?**				
• Yes	6.3	3.5	χ^2^ = 8.33, p < .05	0.07
• No	85.1	88.4		
• Uncertain	8.6	8.1		
**Diagnosed with Lyme disease by a doctor?**				
• Yes	3.3	0.2	χ^2^ = 31.73, *p* < 0.001	0.13^†^
• No	95.9	99.6		
• Uncertain	0.8	0.2		

^a^ The Fisher Exact Test (FET) was used instead of the chi-square test when at least one cell was lower than 5. Unlike the chi-square test, the FET has no formal statistic like chi-square. Thus, we reported the *p *value

^**b**^ Effect size interpretation: ^†^ Small effect, ^††^ Moderate effect, ^†††^Large effect

Next, we performed t-test analyses (see Table 6) on the 27 other variables. Results indicated that there were statistically significant differences for the item of respondents who believed in the possibility of contracting Lyme disease in their municipality (i.e., risk perception), with a higher average in the telephone survey than in the web survey (0.85 vs. 0.77 on a two-point scale; small effect size). Conversely, compared with respondents in the telephone survey, those in the web survey believed that the consequences for their health of contracting Lyme disease (perceived vulnerability) were more serious: 3.61 vs. 3.38 on a four-point scale; small effect size). They also had a more favorable attitude towards the adoption of Lyme disease prevention behaviors in the next year (3.36 vs. 2.98 on a four-point scale; small effect size) and perceived that it would be easier to protect themselves against Lyme disease in the next year (2.97 vs. 2.78 on a four-point scale; small effect size). Respondents to the web survey also perceived more strongly than those who answered the telephone survey that people who were important to them would support their choice if they adopted behaviors to protect themselves against tick bites in the next year (3.53 vs. 3.31 on a four-point scale; small effect size); had a higher intention of adopting protective behaviors in the next year (3.20 vs. 3.13 on a four-point scale; no effect size); and reported having mowed their lawn more frequently (2.96 vs. 2.80 on a 3-point scale; small effect size). Finally, with regard to items relating to clothing, respondents in the web survey reported having worn light-colored clothing less often (2.46 vs. 2.75 on a 5-point scale; small effect size), examined their clothes to avoid bringing ticks into their home less often (1.78 vs. 2.03 on a 5-point scale; small effect size), and put their clothes in the dryer for six minutes to eliminate ticks that may be there less often (1.26 vs. 1.47 on a 5-point scale; small effect size). Weighted means between the telephone and the web survey statistically differed for 19 of these 27 questions. The analysis of the effect size with Cohen’s d showed that 10 of these differences had no effect size and 9 of them had a small effect size. In summary, our results showed that the magnitude of 19 out of 30 items related to Lyme disease was nil and that of 11 out of 30 was small.

**Table 6:** Comparison of the Weighted Mean of the Web Survey with the Telephone Survey on Lyme-Disease-Related Variables

Type of variables	Means		t test	Cohen’s d ^a^
	Web	Phone		
**Knowledge of the disease**				
• Before responding to this survey, had you ever heard of Lyme disease? (0 = No, 1 = Yes)	0.89	0.92	−2.05*	− 0.09
• Lyme disease is transmitted through tick bites (0 = No, 1 = Yes)	0.98	0.98	0.33	0.02
• The first symptom of Lyme disease is generally a red plaque on the skin (0 = No, 1 = Yes)	0.89	0.90	− 0.44	− 0.02
**Risk perception**				
• In your opinion, what is the risk of you contracting Lyme disease in the next year? (1 = Nil to 6 = very high)	3.17	3.00	3.55***	0.16
• Do you believe in the possibility of contracting Lyme disease in your municipality? (0 = No, 1 = Yes)	0.77	0.85	− 4.25****	− 0.21^†^
**Vulnerability**				
• If you were to contract Lyme disease, would you say that the consequences for your health would be very serious? (1 = No, not at all, to 4 = Yes, absolutely)	3.61	3.38	7.60****	0.36^†^
**Opinions about vaccination**				
• If a vaccine against Lyme disease were available, you would get vaccinated. *(1 = Strongly disagree to 4 = Strongly agree)*	3.19	3.09	2.25	0.11
• If a vaccine against Lyme disease were available, you would get your child vaccinated *(1 = Strongly disagree to 4 = Strongly agree)*	3.19	3.03	0.66	0.10
• Vaccines are a danger to your health *(1 = Strongly disagree to 4 = Strongly agree)*	2.08	1.96	2.55*	0.12
**Theory of planned behavior constructs**				
• **Attitudes towards the adoption of preventive behaviors**				
Adopting behaviors to protect yourself against Lyme disease in the next year will be (1 = very useless to 4 = very useful)	3.36	2.98	8.35****	0.39^†^
• **Perceived behavioral control**				
It will be easy to protect yourself against Lyme disease in the next year *(1 = Strongly disagree to 4 = Strongly agree)*	2.97	2.78	5.46****	0.26^†^
• **Perceived social norms**				
If you adopt behaviors to protect yourself against tick bites and therefore Lyme disease in the next year, people who are important to you will support your choice *(1 = Strongly disagree to 4 = Strongly agree)*	3.53	3.31	6.67****	0.31^†^
• **Behavioral intentions**				
You intend to adopt behaviors to protect yourself against tick bites and Lyme disease in the next year *(1 = Strongly disagree to 4 = Strongly agree)*	3.20	3.13	2.19*	0.10
**Preventive behaviors**				
• Have ever looked into ways to prevent Lyme disease for your physical or mental health? (0 = No, 1 = Yes)	0.44	0.40	2.17*	0.10
• Have ever looked into the potential consequences of Lyme disease for your physical or mental health? (0 = No, 1 = Yes)	0.47	0.42	2.38*	0.11
• When practicing outdoor activities, do you wear long pants and a long-sleeved sweater? (1 = never to 5 = always)	3.26	3.12	2.77**	0.13
• When practicing outdoor activities, do you wear closed shoes? (1 = never to 5 = always)	4.00	3.98	0.26	0.01
• When practicing outdoor activities, do you tuck the bottom of your sweater or of your shirt into your pants? (1 = never to 5 = always)	2.18	2.45	− 3.92****	− 0.19
• When practicing outdoor activities, do you tuck the bottom of your pants into your socks or boots? (1 = never to 5 = always)	1.61	1.81	− 3.75***	− 0.18
• When outdoors, do you use a bug repellent (containing DEET, icaridin or picaridin) on your clothes or the exposed parts of your body? (1 = never to 5 = always)	2.69	2.85	− 2.82	− 0.13
• When practicing outdoor activities, do you walk on cleared paths and trails, avoiding tall grass? (1 = never to 5 = always)	3.81	3.98	− 3.31***	− 0.16
• When practicing outdoor activities, do you wear light-colored clothing to make it easier to check for ticks? (1 = never to 5 = always)	2.46	2.75	− 5.45***	− 0.26^†^
• After being outdoors, examine your body for ticks and remove them immediately (1 = never to 5 = always)	2.23	2.29	− 0.89	− 0.04
• After being outdoors, do you examine your clothes and the items that you had with you to avoid bringing ticks into your home? (1 = never to 5 = always)	1.78	2.03	− 4.35****	− 0.21^†^
• After being outdoors, do you put your clothes in the dryer for six minutes to eliminate ticks that may be there? (1 = never to 5 = always)	1.26	1.47	− 4.76****	− 0.23^†^
• Do you regularly mow your lawn or have it mown? (I don’t have a lawn, No, Yes once a week or less, Yes more than once a week	2.96	2.80	5.29****	0.31^†^
• How often do you maintain your lawn, for example pick up dead leaves, weeds, branches or twigs, or have them picked up (other than mowing your lawn)? (Never to more than once a week)	3.24	3.23	0.02	0.001

*p < .05. **p < .01. ***p < .001 ****p < .0001

^**a**^ Effect size interpretation: ^†^ Small effect, ^††^ Moderate effect, ^†††^Large effect

### Measurement invariance

We tested the measurement invariance of the Lyme disease prevention index between the unweighted telephone and web samples. The results showed an adequate fit of the model to the data throughout all the invariance tests, with CFI and TLI > 0.90 and RMSEA < 0.055. The results also supported strong, strict, and latent means invariances across the groups, with ΔCFI and ΔTLI greater than − 0.01 and ΔRMSEA not exceeding + 0.015 in both cases. The results did not support variance invariance across the groups, with ΔRMSEA not exceeding + 0.015 but ΔCFI and ΔTLI exceeding the threshold of − 0.010 (− 0.022 and − 0.021, respectively). Overall, the results showed invariance of the Lyme disease prevention index across the telephone and web samples.

The results of the invariance tests for the weighted data are displayed in parentheses in Table 7. The results showed an excellent fit of the model to the data throughout all the tests, with CFI and TLI > 0.90 and RMSEA < 0.035. There was also strong, strict, and latent means invariance across the groups, with ΔCFI and ΔTLI greater than − 0.01 and ΔRMSEA not exceeding + 0.015 in both cases. No latent variance invariance was observed here, with ΔRMSEA not exceeding + 0.015 but ΔCFI and ΔTLI exceeding the threshold of − 0.010 (both equal to − 0.023). Then, there is also invariance of the Lyme disease prevention index for the weighted data.

**Table 7:** Measurement Invariance of the Index of Adaptation

Model	χ^2^	df	RMSEA	CFI	TLI	ΔRMSEA	ΔCFI	ΔTLI	Compared Model
Configural invariance	233.558 (137.077)	70	0.049 (0.031)	0.945 (0.956)	0.930 (0.944)	–	–	–	–
Strong invariance	235.534 (136.762)	78	0.045 (0.028)	0.947 (0.962)	0.939 (0.956)	−0.004 (− 0.003)	0.002 (0.006)	0.009 (0.012)	1
Strict invariance	272.407 (154.383)	88	0.046 (0.028)	0.939 (0.957)	0.937 (0.956)	0.001 (0)	−0.008 (−0.005)	− 0.002 (0)	2
Variance invariance	337.880 (190.946)	89	0.054 (0.034)	0.917 (0.934)	0.916 (0.933)	0.008 (0.006)	−0.022 (− 0.023)	−0.021 (− 0.023)	3
Latent means invariance	330.308 (188.329)	90	0.052 (0.033)	0.920 (0.936)	0.920 (0.936)	−0.002 (− 0.001)	0.003 (0.002)	0.004 (0.003)	4

## Discussion

Our first objective was to compare the representativeness of the non-probabilistic web sample and the probabilistic telephone sample with census data in terms of sociodemographic characteristics (gender, age, highest education level, annual income, presence of at least one child in the household, household size). Our results showed that neither survey modes could be considered more representative of the Canadian census based on these demographic characteristics. This finding is not in line with the majority of past findings suggesting that probability samples are more representative of national data than non-probability samples [e.g. [22, 67–69] but it is in line with a few other ones [e.g. [37].

To put it plainly, regarding the representativeness of the unweighted samples produced through the two contact methods in terms of certain characteristics of the population described in the census data, our results indicated some differences but did not enable us to qualify one approach as being superior to the other. The fact that more and more people have access to the Internet today, which makes it possible to reach a larger number of people who are older, live in remote regions, and are of lower socioeconomic levels [37], certainly contributes to largely attenuating certain deficiencies in representativeness revealed in previous years. Our data clearly showed some differences. For example, the proportion of respondents between 18 and 34 years old was higher and was closer to the proportion observed in the census in the web survey than in the telephone survey (probably due to the advent of smartphones). Nonetheless, it remained much smaller than that observed in the census. Furthermore, the proportion of elderly people (75 years and over) in the web survey did not differ statistically from that in the census, contrary to what might have been expected. Inversely, the proportion of people of whom at least one child lived at home in the telephone survey was closer to that in the census.

Our results also showed that the magnitude of the demographic differences in response rates between the two unweighted samples (telephone and web) was nil, according to the effect size analyses. Household income is often associated with health inequity, so this question should always be included in health questionnaires. However, its non-response rate is usually high [70]. Our study was no exception, with a non-response rate for household annual gross income above 15%. Although the effect size was nil, the non-response rate was lower in the web survey than in the telephone survey.

The second objective was to compare both survey estimates in regard to reported Lyme disease adaptive behaviors and other theoretically associated variables (e.g. knowledge of Lyme disease, risk perception, attitudes). Our findings showed that there were more item non-responses in the web survey than in the telephone survey (i.e. 12 small, one medium, and 17 nil effect sizes). This result can be explained by the fact that a “don’t know” option was explicitly offered in the web survey but was not read by the interviewer in the telephone survey. If this “don’t know” option is not offered to respondents, they tend to quit [71, 72]. Consequently, we decided to offer this answer choice for the appropriate questions. For instance, for the question “Do you believe in the possibility of contracting Lyme disease in your municipality,” 27.3% of respondents in the web survey chose “don’t know,” compared with only 4.6% of respondents in the telephone survey (even if the “don’t know” option was not explicitly offered in the latter survey mode). The same tendency was observed for four questions of a psychosocial nature. These questions all referred to the respondents’ opinions on the possibility of their adopting preventive behaviors in the “next year.” Those questions (attitude, perceived control, perceived social norms, intention) were all based on the theory of planned behavior [73]. It may be difficult for respondents to know what they will do in the next year. It is thus not surprising that the respondents in the web survey chose the “don’t know” option more often because it was explicitly offered to them, contrary to in the telephone survey. However, for the self-reported preventive behaviors, the non-response rates were lower for both survey modes. One potential hypothesis is that the higher item non-response rate observed for the web survey had no impact on the survey estimates. In fact, on the 13 out of 30 items showing higher non-response levels for the web survey (i.e. statistical difference and small effect size), half showed estimate differences with the telephone survey (for the weighted and unweighted data).

Furthermore, the results of our study show that both survey modes provided similar estimates of the adoption of various behaviors for protecting against Lyme disease or of their determinants (e.g. exposure to Lyme disease, knowledge, opinions on vaccination, risk perception). Besides those previously mentioned, another factor may explain this finding: the low response rates in the two data collections (24 and 17% in the telephone and web surveys, respectively). Yet, such percentages are not surprising, given that research has demonstrated that response rates have continuously dropped over the past 30 years [37, 74–77]. People are increasingly asked to participate in surveys, both by phone and on the web, and the consequence is a decrease felt in their interest in responding. With caller ID, it is now possible to spot suspicious phone numbers and to easily avoid participating in a telephone survey. The low response rates observed for each collection mode does not preclude the hypothesis that the non-response bias was high in both cases [75]. Weighting the data to match the proportions in the Canadian census with respect to the sociodemographic variables and obtaining statistical estimates of the same magnitude did not make it possible to estimate the extent of a potential non-response bias in either case.

The third goal of this study was to test the measurement invariance or equivalence of the latent construct of Lyme disease adaptive behaviors across the non-probabilistic web panel and telephone samples. Our results showed that there was metric and latent mean invariance of the behavioral index for adaptation to Lyme disease. Thus, it appears that the structure of the index remained the same for both survey modes. Second, the latent means invariance observed indicated that the levels of adaptation measured in the web and telephone surveys did not differ significantly from one another. In other words, a level of adaptation estimated from a composite index would be the same whether estimated from a web survey or from a telephone survey. These results are encouraging and also confirm that the behavioral index for adaptation to Lyme disease is robust across both survey modes. In summary, the index was considered equivalent whether measured by a web questionnaire or a telephone survey, even though the web data had much more occurrences of item non-response. In other words, our data showed that even though the web mode has a higher rate of item non-response than the telephone mode, this has very little effect on the estimated parameters. This is an important contribution because, as far as we know, no study has shown the invariance of an index across the non-probabilistic web panels and telephone samples.

Some researchers might still argue that a larger sample size may be needed when using the web panel mode of data collection rather than the telephone mode because it has a higher rate of item non-response. However, since web surveys are typically three times cheaper, this should not be problematic.

In the case of our study in particular, we faced some limitations during the data collection process, particularly with the web survey. Despite extended Internet networks, some of the smallest municipalities were still more difficult to contact through the web survey, because of a lower Internet reach in those areas. Therefore, the quotas initially fixed for some regions were not met. Moreover, it is possible that the non-response bias (i.e., bias resulting from respondents differing considerably from non-respondents) was high in both survey modes. In fact, as suggested by Tourangeau [72], there is a significant relationship between non-response rates and non-response bias.

## Conclusions

A number of interesting outcomes from this study led us to believe that the representativeness and estimates obtained with web and telephone surveys would be similar, at least if a study in which the respondents’ answers would not be affected by the tendency to want to look good socially (social desirability bias). Thus, in studies where adaptation to climate change is monitored over time, using a web survey instead of a telephone survey could be more cost-effective (about a third of the price; in our study, CAD 18,200 and CAD 7800 for telephone and web surveys, respectively), and we will not hesitate to do so in our next surveys on adaptation to climate change [29, 30, 41, 78, 79]. That being said, we should nevertheless keep in mind the disadvantages of a web survey.

Finally, two things remain certain. First, multiplying between-group comparisons always leads to the observation or detection of differences attributable to uncertain factors, sources or phenomena. Furthermore, from the moment people refuse to participate in a survey or to respond to certain questions, a sample loses its representativeness, and the results risk being tainted by various sampling and non-response biases. The fact remains, however, that the results of our study did not provide the information needed to determine that one survey mode is superior to the other.

## Supplementary information

**Additional file 1: Online resource 1.** Quotas originally provided to the polling firm. **Online resource 2.** Samples’ actual numbers per region. **Online resource 3.** Socio-demographic characteristics: comparing weighted web/phone samples and census. **Online resource 4.** Comparison of the unweighted item non-responses of the Web and Telephone Surveys on Lyme Disease Exposure.

## Data Availability

The datasets used and/or analysed during the current study are available from the corresponding author on reasonable request.

## Abbreviations

RDD: Random digit dialing
LDPB: Lyme disease prevention behaviors
CFI: Comparative fit index
TLI: Tucker-Lewis index
RMSEA: Root mean squared error of approximation
FET: Fisher exact tests
