# Supplementary material for: Telephone versus web panel National Survey for monitoring adoption of preventive behaviors to climate change in populations: a case study of Lyme disease in Québec, Canada
Source: BMC Med Res Methodol. 2020 Apr 10;20:78. doi: 10.1186/s12874-020-00958-4 (PMC7146908; doi:10.1186/s12874-020-00958-4)
Supplement: Supplementary file 1 — Additional file 1: Online resource 1. Quotas originally provided to the polling firm. Online resource 2. Samples’ actual numbers per region. Online resource 3. Socio-demographic characteristics: comparing weighted web/phone samples and census. Online resource 4. Comparison of the unweighted item non-responses of the Web and Telephone Surveys on Lyme Disease Exposure. [file 12874_2020_958_MOESM1_ESM.docx]

# Additional files

**Online resource 1.** Quotas originally provided to the polling firm

| Region | Web sample | Telephone sample |
| --- | --- | --- |
| Montérégie | 200 | 200 |
| Estrie | 185 | 185 |
| Mauricie-et-Centre-du-Québec | 100 | 100 |
| Outaouais | 100 | 100 |
| Lanaudière | 80 | 80 |
| Laurentides | 80 | 80 |
| Chaudière-Appalaches | 55 | 55 |
| Montréal | 100 | 100 |
| Laval | 100 | 100 |
| Total | 1000 | 1000 |

**Online resource 2.** Samples’ actual numbers per region

| Region | Web sample | Telephone sample |
| --- | --- | --- |
| Montérégie | 208 | 207 |
| Estrie | 152 | 182 |
| Mauricie-et-Centre-du-Québec and Chaudière-Appalaches | 130 | 154 |
| Outaouais | 103 | 99 |
| Lanaudière | 79 | 79 |
| Laurentides | 80 | 82 |
| Montréal | 103 | 101 |
| Laval | 101 | 99 |
| Total | 956 | 1003 |

**Online resource 3.** Socio-demographic characteristics: comparing weighted web/phone samples and census

| Socio-demographic variables | Census | Sample | | Chi-square test (χ^2^) and effect size (Cramer’s V) | | | | | |
| --- | --- | --- | --- | --- | --- | --- | --- | --- | --- |
|  |  | Web | Phone | Web-Census | | Phone-Census | | Web-Telephone | |
|  |  |  |  | χ^2^ | Cramer’s V | χ^2^ | Cramer’s V | χ^2^ | Cramer’s V |
| **Age** |  |  |  |  |  |  |  |  |  |
| - 18 - 34 | 25.6% | 25.5% | 25.6% | 0.04 | 0.00 | 0.09 | 0.00 | 0.01 | 0.00 |
| - 35 - 44 | 16.4% | 16.4% | 16.2% |  |  |  |  |  |  |
| - 45 - 54 | 18.8% | 18.6% | 18.7% |  |  |  |  |  |  |
| - 55 - 64 | 18.5% | 18.7% | 18.7% |  |  |  |  |  |  |
| - 65 - 74 | 12.9% | 13.1% | 13.1% |  |  |  |  |  |  |
| - 75 and more | 7.7% | 7.7% | 7.7% |  |  |  |  |  |  |
| **Gender** |  |  |  |  | |  | |  |  |
| - Male | 48.5% | 48.6% | 48.6% | 0.00 | 0.00 | 0.00 | 0.00 | 0.00 | -0.00 |
| - Female | 51.5% | 51.4% | 51.4% |  |  |  |  |  |  |
| **Highest education level** |  |  |  |  | |  | |  |  |
| - No certificate, diploma or degree | 17.4% | 17.8% | 17.6% | 0.33 | 0.01 | 0.27 | 0.01 | 0.03 | 0.00 |
| - Secondary (high) school diploma or equivalency certificate | 23.3% | 23.5% | 23.4% |  |  |  |  |  |  |
| - Diploma or certificate of college, trade or vocational studies, partial university studies | 39.3% | 39.4% | 39.7% |  |  |  |  |  |  |
| - University degree | 20.0% | 19.3% | 19.3% |  |  |  |  |  |  |
| **Household size** |  |  |  |  | |  | |  |  |
| - 1 person | 30.5% | 30.1% | 30.8% | 0.15 | 0.01 | 0.10 | 0.01 | 0.11 | 0.01 |
| - 2 persons | 34.2% | 34.7% | 34.4% |  |  |  |  |  |  |
| - 3 persons and more | 35.3% | 35.2% | 34.8% |  |  |  |  |  |  |
| **Children in the household** |  |  |  |  | |  | |  |  |
| - Yes | 39.2% | 38.9% | 37.7% | 0.04 | 0.01 | 0.95 | 0.03 | 0.29 | 0.01 |
| - No | 60.8% | 61.1% | 62.3% |  |  |  |  |  |  |
| **Household annual gross income** |  |  |  |  | |  | |  | |
| - 20 000$ and less | 9.7% | 9.6% | 10.8% | 0.05 | 0.00 | 7.04 | 0.05 | 4.21 | 0.05 |
| - 20 001$ - 60 000$ | 37.1% | 37.3% | 37.3% |  |  |  |  |  |  |
| - 60 001$ - 100 000$ | 26.3% | 26.1% | 28.8% |  |  |  |  |  |  |
| - More than 100 000$ | 26.9% | 27.0% | 23.1% |  |  |  |  |  |  |

*p<.05. **p<.01. ***p<.001 ****p<.0001

Effect size interpretation: ^†^ Small effect, ^††^ Moderate effect, ^†††^large effect

**Online resource 4.** Comparison of the unweighted item non-responses of the Web and Telephone Surveys on Lyme Disease Exposure

| Type of variables | Unweighted item non-responses (%) | | χ^2^ test or  Fisher exact test (FET)^a^ | Cramer’s V^b^ |
| --- | --- | --- | --- | --- |
|  | Web | Phone |  |  |
| **Found a tick on yourself?** | 2.2 | 0.3 | P < .0001; FET | -0.09 |
| - Yes |  |  |  |  |
| - No |  |  |  |  |
| - Uncertain |  |  |  |  |
| **Ever been bitten by a tick?** | 5.2 | 0.4 | P < .0001; FET | -0.15^†^ |
| - Yes |  |  |  |  |
| - No |  |  |  |  |
| - Uncertain |  |  |  |  |
| **Diagnosed with**  **Lyme disease by**  **a doctor?** | 1.2 | 0.1 | χ^2^ = 8.88, p < .01 | -0.07 |
| - Yes |  |  |  |  |
| - No |  |  |  |  |
| - Uncertain |  |  |  |  |
| **Knowledge of the disease** |  |  |  |  |
| - Before responding to this survey, had you ever heard of Lyme disease?   (0 = No, 1 = Yes) | 1.6 | 0.0 | P < .0001; FET | -0.09 |
| - Lyme disease is transmitted through tick bites (0 = No, 1 = Yes) | 3.5 | 1.4 | 8.84** | -0.07 |
| - The first symptom of Lyme disease is generally a red plaque on the skin   (0 = No, 1 = Yes) | 18.4 | 8.4 | 42.82**** | -0.15^†^ |
| **Risk perception** |  |  |  |  |
| - In your opinion, what is the risk of you contracting Lyme disease in the next year? (1 = Nil to 6 = very high) | 8.2 | 1.9 | 40.82**** | -0.14^†^ |
| - Do you believe in the possibility of contracting Lyme disease in your municipality? **(**0 = No, 1 = Yes) | 27.3 | 4.6 | 191.10**** | -0.31^††^ |
| **Vulnerability** |  |  |  |  |
| - If you were to contract Lyme disease, would you say that the consequences for your health would be very serious?   (1 = No, not at all, to 4 = Yes, absolutely) | 8.1 | 3.5 | 18.92**** | -0.10^†^ |
| **Opinions about vaccination** |  |  |  |  |
| - If a vaccine against Lyme disease were available, you would get vaccinated. *(1 = Strongly disagree to 4 = Strongly agree)* | 16.0 | 1.9 | 121.67**** | 0.25^†^ |
| - If a vaccine against Lyme disease were available, you would get your child vaccinated *(1 = Strongly disagree to 4 = Strongly agree)* | 0.8 | 0.3 | P = .14; FET | -0.04 |
| - Vaccines are a danger to your health   *(1 = Strongly disagree to 4 = Strongly agree)* | 12.9 | 4.4 | 45.13**** | -0.15^†^ |
| **Theory of planned behavior constructs** |  |  |  |  |
| - **Attitudes towards the adoption of preventive behaviors** |  |  |  |  |
| Adopting behaviors to protect yourself against Lyme disease in the next year will be (1 = very useless to 4 = very useful) | 6.9 | 0.5 | 57.49**** | -0.17^†^ |
| - **Perceived behavioral control** |  |  |  |  |
| It will be easy to protect yourself against Lyme disease in the next year  *(1 = Strongly disagree to 4 = Strongly agree)* | 15.7 | 3.6 | 83.411**** | -0.21^†^ |
| - **Perceived social norms** |  |  |  |  |
| If you adopt behaviors to protect yourself against tick bites and therefore Lyme disease in the next year, people who are important to you will support your choice  *(1 = Strongly disagree to 4 = Strongly agree)* | 9.6 | 1.6 | 60.56**** | 0.18^†^ |
| - **Behavioral intentions** |  |  |  |  |
| You intend to adopt behaviors to protect yourself against tick bites and Lyme disease in the next year  *(1 = Strongly disagree to 4 = Strongly agree)* | 8.5 | 0.7 | 68.96**** | -0.19^†^ |
| **Preventive behaviors** |  |  |  |  |
| - Have ever looked into ways to prevent Lyme disease for your physical or mental health? (0 = No, 1 = Yes) | 2.9 | 0.4 | P < .0001; FET | -0.10^†^ |
| - Have ever looked into the potential consequences of Lyme disease for your physical or mental health? (0 = No, 1 = Yes) | 1.9 | 0.1 | P < .0001; FET | -0.09 |
| - When practicing outdoor activities, do you wear long pants and a long-sleeved sweater? (1 = never to 5 = always) | 0.7 | 0.7 | 0.0081 | -0.00 |
| - When practicing outdoor activities, do you wear closed shoes? (1 = never to 5 = always) | 0.6 | 0.2 | P = .017; FET | -0.03 |
| - When practicing outdoor activities, do you tuck the bottom of your sweater or of your shirt into your pants? (1 = never to 5 = always) | 0.6 | 0.1 | P = .06; FET | -0.04 |
| - When practicing outdoor activities, do you tuck the bottom of your pants into your socks or boots? (1 = never to 5 = always) | 1.3 | 0.4 | P = .04; FET | -0.05 |
| - When outdoors, do you use a bug repellent (containing DEET, icaridin or picaridin) on your clothes or the exposed parts of your body?   (1 = never to 5 = always) | 1.6 | 0.6 | 4.35* | -0.05 |
| - When practicing outdoor activities, do you walk on cleared paths and trails, avoiding tall grass? (1 = never to 5 = always) | 2.2 | 0.5 | 10.78** | -0.07 |
| - When practicing outdoor activities, do you wear light-colored clothing to make it easier to check for ticks? (1 = never to 5 = always) | 4.8 | 0.5 | 35.91**** | -0.14^†^ |
| - After being outdoors, examine your body for ticks and remove them immediately (1 = never to 5 = always) | 1.9 | 0.5 | 8.08** | -0.06 |
| - After being outdoors, do you examine your clothes and the items that you had with you to avoid bringing ticks into your home?   (1 = never to 5 = always) | 1.6 | 0.2 | P < .0001; FET | -0.07 |
| - After being outdoors, do you put your clothes in the dryer for six minutes to eliminate ticks that may be there? (1 = never to 5 = always) | 1.6 | 0.2 | P < .0001; FET | -0.08 |
| - Do you regularly mow your lawn or have it mown?   (I don’t have a lawn, No, Yes once a week or less, Yes more than once a week | 1.0 | 1.6 | 0.96 | 0.03 |
| - How often do you maintain your lawn, for example pick up dead leaves, weeds, branches or twigs, or have them picked up (other than mowing your lawn)? (Never to more than once a week) | 0.9 | 0.8 | 0.12 | -0.01 |

^a^ The Fisher Exact Test (FET) was used instead of the chi-square test when at least one cell was lower than 5. Unlike the chi-square test, the FET has no formal statistic like chi-squared. Thus, we reported the p value.

**^b^** Effect size interpretation: ^†^ Small effect, ^††^ Moderate effect, ^†††^Large effect
